# Comparing Airborne Particulate Matter Intake Dose Assessment Models Using Low-Cost Portable Sensor Data

**DOI:** 10.3390/s20051406

**Published:** 2020-03-04

**Authors:** Rok Novak, David Kocman, Johanna Amalia Robinson, Tjaša Kanduč, Dimosthenis Sarigiannis, Milena Horvat

**Affiliations:** 1 Department of Environmental Sciences, Jožef Stefan Institute, 1000 Ljubljana, Slovenia; david.kocman@ijs.si (D.K.); johanna.robinson@ijs.si (J.A.R.); tjasa.kanduc@ijs.si (T.K.); milena.horvat@ijs.si (M.H.); 2 Jožef Stefan International Postgraduate School, 1000 Ljubljana, Slovenia; 3 Environmental Engineering Laboratory, Department of Chemical Engineering, Aristotle University of Thessaloniki, 54124 Thessaloniki, Greece; denis@eng.auth.gr; 4 HERACLES Research Centre on the Exposome and Health, Center for Interdisciplinary Research and Innovation, 54124 Thessaloniki, Greece; 5 University School of Advanced Study IUSS, 27100 Pavia, Italy

**Keywords:** dose assessment, particulate matter, minute ventilation, low-cost sensors, uncertainty assessment

## Abstract

Low-cost sensors can be used to improve the temporal and spatial resolution of an individual’s particulate matter (PM) intake dose assessment. In this work, personal activity monitors were used to measure heart rate (proxy for minute ventilation), and low-cost PM sensors were used to measure concentrations of PM. Intake dose was assessed as a product of PM concentration and minute ventilation, using four models with increasing complexity. The two models that use heart rate as a variable had the most consistent results and showed a good response to variations in PM concentrations and heart rate. On the other hand, the two models using generalized population data of minute ventilation expectably yielded more coarse information on the intake dose. Aggregated weekly intake doses did not vary significantly between the models (6–22%). Propagation of uncertainty was assessed for each model, however, differences in their underlying assumptions made them incomparable. The most complex minute ventilation model, with heart rate as a variable, has shown slightly lower uncertainty than the model using fewer variables. Similarly, among the non-heart rate models, the one using real-time activity data has less uncertainty. Minute ventilation models contribute the most to the overall intake dose model uncertainty, followed closely by the low-cost personal activity monitors. The lack of a common methodology to assess the intake dose and quantifying related uncertainties is evident and should be a subject of further research.

## 1. Introduction

Application of low-cost air quality (AQ) sensors is on the rise and is being used to determine air pollution in cities [1,2,3,4], monitoring of indoor AQ [5,6,7], and for exposure assessment [8,9,10]. Traditionally exposure studies use data from monitoring stations, questionnaires, or biomarkers [11], and more recently land-use regression models [12,13] and other modelling techniques [14], while the most sought-after method is measuring intake dose on a personal level [15]. To this end, low-cost sensors that have become smaller and more energy-efficient, and now enable subjects to carry these devices with them, can significantly improve the temporal and spatial resolution of information needed [16]. However, although continuous and rapid advances in sensing technologies are resulting in improved accuracy, these devices still need extra validation and/or calibration before being put to use [9,17,18]. They could employ a wide array of options to achieve more accurate results, such as comparing with reference analysers [8] or using sophisticated artificial intelligence approaches [19]. On the other hand, assessing intake dose is not only dependent on the concentrations of pollutants, but also other factors, mainly a person’s breathing rate or ventilation [20]. Several studies throughout the past three decades have shown that minute ventilation is corelated with heart rate [21,22,23,24]. Ventilation can be estimated by various approaches and models, which differ mostly by the number and type of variables used, from more generalized approaches using sex, age, and ethnicity [25] with different kinds of activities [26], to more specific and complex models with additional variables such heart rate [27], forced vital capacity and breath frequency [28], and hip circumference [29]. All of these approaches do offer some advantages, often as trade-offs to accuracy. Less complex approaches use personal information, such as age and sex, and determine minute ventilation from generalized population data [25,30], while more complex models use continuously monitored variables, such as heart rate (HR) [27,28].

The aims of this study are as follows:

- to evaluate the applicability of different intake dose models by assessing the uncertainty associated with each input variable;
- to estimate how the uncertainty propagates forward and affects the uncertainty of the model;
- to compare the results calculated with the models on two contrasting individuals;
- to evaluate the complexity of the models, time, and resource requirements and the burdens participants have in providing the data.

In this work, four different approaches to assess the PM intake dose are compared, using data obtained by two participants included in the sampling campaign conducted within the ICARUS H2020 project [31], which was separated into winter (February) and summer (May) campaigns, and took place in the first half of 2019. The participants carried a portable PM sensor and a heart rate monitor with them at all times and measured indoor and outdoor concentrations of PM during the entire seven-day period.

The uncertainty associated with each intake dose assessment model was quantified and the hypothesis was that the less complex models would provide data with more uncertainty, as they use variables that have higher uncertainties and are based on more generalizations (e.g., average minute ventilation for a 60-year-old female in a less complex model, in contrast with minute ventilation derived directly from measured heart rate in more complex models). Propagation of uncertainty from low-cost sensors and minute ventilation models to intake dose assessments was investigated. A crucial component of the overall uncertainty assessment is to determine the validity of the PM concentration data from the low-cost sensor. To this end, the performance of the low-cost PM sensor was evaluated by collocating it with a reference instrument in an office environment. Moreover, uncertainties calculated and presented for each minute ventilation model were not consistent from paper to paper, as was the nomenclature regarding exposure science and metrology. These issues are addressed and discussed.

## 2. Materials and Methods

### 2.1. Terminology and Nomenclature

Terminology and nomenclature used in this work are based on the following sources. Terms regarding the human–pollutant interaction (e.g., “personal exposure”, “intake dose”, “exposure assessment”) were adopted from the Official International Society for Exposure Analysis glossary by Zartarian et al. [31]. Terms related to metrology and statistics (e.g., “uncertainty”, “reproducibility”, “validity”) were adopted from the International Vocabulary of Metrology [32].

### 2.2. Measuring Particulate Matter Concentrations

A portable Arduino based low-cost PM measuring unit (referred to as PPM) was developed for the ICARUS project [33] by IoTech Telecommunications, Thessaloniki, Greece [34], and used in this research. Using Plantower, Beijing, China, pms5003 sensor [35], based on the laser light scattering principle, the PPM unit provides data for concentrations of PM in one-minute resolution.

PM concentration data are provided in three size classes/channels: <1 µm (PM_1_), <2.5 µm (PM_2.5_), and <10 µm (PM_10_). A detailed description of the instrument with specifications is provided in the Supplementary Materials.

Weekly and daily averages of PM_2.5_ and PM_10_ measurements from the collocation and personal monitors were additionally compared with values obtained from the government run AQ station, near the centre of Ljubljana (2 km from where the collocation took place), to determine if the values were close. The averages were compared to determine if the values measured by the sensors were in the same range as those measured at the AQ station.

#### Collocation of the PPM Unit with a Reference Instrument

The PPM unit was collocated with a GRIMM (Durag Group, Hamburg, Germany) Model 11-A (1.109) Aerosol Spectrometer (GRIMM), which was used as a research-grade reference instrument for PM measurements. The collocation lasted one week, from 5 to 12 March 2019, and was located at an office space with open windows at Jožef Stefan Institute, Ljubljana, Slovenia (LAT: 14.4879, LON: 46.0424). The PPM unit provided data with minute resolution and GRIMM with five-minute resolution. Data recovery for the measuring period was 100% for the PPM unit and 99.8% for GRIMM. A more detailed description of the instrument is available in the Supplementary Materials.

There was a four-hour period with light precipitation during the collocation, the mean (min – max) temperature throughout the week was 12.3°C (6.3°C–16.3°C) and relative humidity was 55% (36–65%).

### 2.3. Measuring Heart Rate and Physical Activity

Continuous HR measurements were made by using a commercial smart activity tracker (SAT), a Vívosmart 3 from Garmin International [36]. The data were in minute temporal resolution and provided values for HR, specific physical activity, steps taken, calories “burned”, distance walked, and stress.

Uncertainty of SAT was estimated based on the work of Oniani et al. [37], who compared the same device (Garmin Vivosmart 3) with an ECG (electrocardiogram). In their work, four participants were selected, and equipped with four SAT devices each. An 80-min treadmill test was performed with each participant, with four different speeds of walking, while being connected to an ECG monitor. The results were presented with MAPE (mean absolute percentage error) and ICC (intraclass correlation) for each device, in comparison to the ECG.

### 2.4. Calculating Personal Intake Dose of Airborne Particulate Matter

Intake dose of PM was calculated as a product of PM concentration, in this case, PM_1_ concentrations for one-minute average values (as the results of the collocation showed, this proved to be the measurement with the lowest uncertainty associated with it), measured with a PPM device, and minute ventilation data, which was determined by four different models described below. The intake dose model (M1–M4) calculation is presented in Equation (1):

(1) $$intake\;dose=\;{\dot{V}}_{E}*PM_{1},$$

where ${\dot{V}}_{E}$ presents minute ventilation (L min^-1^) and *PM_1_* is the particulate matter concentration measured with the PPM sensor (µg m^-3^).

Two of the described models (M1 and M2) use HR as a variable, and two (M3 and M4) do not, and in turn use average minute ventilation data for specific population groups. Comparison between the four models was performed based on the data from two participants involved in the ICARUS personal exposure assessment campaign in Ljubljana:

- P1: 60-year-old Caucasian female participant, weighing 62 kg, height 166 cm. P1 participated in the campaign between 17 and 23 February 2019, and was located in Ljubljana the entire period. P1 was employed as an office worker.
- P2: 35-year-old Caucasian male participant, weighing 66 kg, height 178 cm. P2 participated in the campaign between 14 and 21 May 2019, and was also located in Ljubljana the entire period. P2 was employed as a bike courier.

Using only one participant could skew the results, including another participant with a contrasting profile (different personal characteristics, such as sex, height, and age) enables a more thorough comparison. Two participants are enough for the purposes of this research, as the goal is to compare models and not validate them for larger groups.

#### 2.4.1. Model 1 (M1)

Minute ventilation model in M1 (${\dot{V}}_{E}^{1}$) is based on the work of Greenwald et al. [28], who modelled minute ventilation with HR, age, sex, and forced vital capacity (FVC) as variables, with the explicit goal to use these data in pollution intake dose estimates. Data from 471 subjects from 8 different studies were compiled in their research and enabled the researchers to gather a dataset of 14,550 one-minute data points. Here, their best performing model was selected, using HR data, combined with information about the subject’s sex, age, height, and weight (used in determining FVC):

(2) $${\dot{V}}_{E}^{1}=e^{-9.59}HR^{2.39}age^{0.274}sex^{-0.204}FVC^{0.520},$$

where ${\dot{V}}_{E}^{1}$ presents minute ventilation for M1; *age* is the age of the participant in years; *sex* is the participants sex, where value 1 is male and 2 is female; and *FVC* is forced vital capacity.

FVC factor was estimated using the Global Lung Function Initiative methodology [38]. FVC for P1 was 3.32 l (lower limit at 2.61 l, upper at 4.16 l, for a 90% confidence interval) and for P2 5.37 l (lower: 4.30 l, upper: 6.45 l, for a 90% confidence interval).

M1 is calculated based on Equation (1).

#### 2.4.2. Model 2 (M2)

${\dot{V}}_{E}^{2}$ is based on the work of Zuurbier et al. [27], who used a simplified approach with only HR and sex as variables:

(3) $${\dot{V}}_{E}^{2}={\left(a*HR+b\right)}^{e},$$

where ${\dot{V}}_{E}^{2}$ presents minute ventilation for M2, HR stands for heart rate, and a and b present the slope and intersect based on sex (a is 0.023 and 0.021, b is 0.57 and 1.03, for females and males, respectively). Their model is based on a study performed with 34 participants.

M2 is calculated based on Equation (1).

#### 2.4.3. Model 3 (M3)

${\dot{V}}_{E}^{3}$ follows an approach by Sarigiannis et al. [25], modelling mercury intake by combining age and ethnicity-specific data of activity patterns, inhalation rates, and body weight, with a specific type of microenvironment. Madureira et al. [30] use a similar approach for indoor intake dose of bioaerosol particles. Each observation is multiplied with a breathing rate factor corresponding to the (hourly) activity self-reported by the participant, but the model does not use any continuous variable, such as HR.

Following the described methods [25,30], in this research, activity reported by the participant in a time activity diary (TAD) was differentiated into four groups, as listed in the U.S. Environmental Protection Agency (EPA) Exposure Factors Handbook [39]: sedentary and passive activities (includes sleep, nap, resting, working behind a desk, and watching TV), light intensity activities (cleaning, cooking), moderate intensity activities (walking, working in garden), and heavy intensity activities (sports, hard manual labour). Average minute ventilation was provided by this handbook for each type of activity, differentiated by age, sex, and body weight. ${\dot{V}}_{E}^{3}$ uses this information to determine minute ventilation for each hourly interval.

M3 is calculated based on Equation (1).

#### 2.4.4. Model 4 (M4)

${\dot{V}}_{E}^{4}$ uses one of the most basic approaches to determine minute ventilation using only a few general data points for the subject: age, sex, and weight. The EPA Exposure Factors Handbook, which provides estimated minute ventilation according to the mean values determined for specific groups [39], also provides generalized data for time spent in micro-locations, doing specific activities for each (age and sex) group. With this information, using Equation (4), it is possible to calculate average minute ventilation, weighted for per cent of time spent doing each activity.

(4) $${\dot{V}}_{E}^{4}\;=\left(sP*aV_{P}+sLi*aV_{Li}+sMi*aV_{Mi}+sHi*aV_{Hi}\right)*BW,$$

where ${\dot{V}}_{E}^{4}$ is minute ventilation for M4; and *sP*, *sLi*, *sMi*, and *sHi* present daily shares of time spent doing *P*—passive, *Li*—light intensity, *Mi*—moderate intensity, and *Hi*—high-intensity activities, respectively. The *aV* factors present average ventilations for that specific activity, according to age group and sex. Factor *BW* is the subjects body weight, which must be included because the ${\dot{V}}_{E}$ data in the EPA handbook are presented in “per kg of body weight” form.

M4 is calculated based on Equation (1).

### 2.5. Statistical Analysis and Determining the Uncertainty

After collocating PPM with the GRIMM, to determine the validity, a Wilks–Shapiro test was conducted for each time-averaging interval to numerically determine normality and a q–q plot was made to visually determine normality. The distribution was non-normal, which prompted the use of the Spearman rank-order correlation test. For each comparison, a scatter plot was made with a linear regression line and 95% confidence interval. RMSE (root mean square error), MAPE (mean absolute percentage error), MAE (mean absolute error), R^2^, slope, and intercept values were calculated.

Summary statistics were calculated for all four models, PM_1_ concentrations, and HR, and for the results iterating M1 over different heights and weights, as presented in the Supplementary Data.

The uncertainty for the PPM was estimated by collocating it with the GRIMM, which has a reproducibility of 5% for the whole range [40]. This measure is carried forward to the PPM device through the collocation process, which enabled the calculation of several statistical measures of agreement (R^2^, MAPE, MAE, RMSE, MAE%). According to the GRIMM manual description, the MAPE measure is the closest, and the uncertainty from the GRIMM is carried forward through the following equation:

(5) $$u\left(PPM\right)=\;\sqrt{u{\left(Grimm\right)}^{2}+u{\left(comparison\right)}^{2}},$$

where *u* is the uncertainty.

All models used in this research had some measure of agreement listed in their evaluation. Not all measures were the same, which makes some of the results incomparable between the models.

${\dot{V}}_{E}^{1}$, from Greenwald et al. [28], had the uncertainty expressed as “per cent error”, which is “the difference between predictions and observations from cross-validation”, and in IQR (interquartile range). The median (IQR) per cent error was −0.664 (45.4)% [28]. With the Supplementary Data, provided by the authors of the paper, it was possible to calculate other statistical measures (R^2^, MAPE, MAE, RMSE). This calculated uncertainty presupposes that all the variables used are categorical (in the cases of sex and weight, this is correct) and without uncertainty. This is not the case in this research, where the SAT device has some uncertainty associated with it, as does the FVC value, with both having different exponents in the model (2.39 for HR and 0.52 for FVC). To determine the overall uncertainty of the model, all the component uncertainties were combined.

${\dot{V}}_{E}^{2}$, based on Zuurbier et al. [27], had its uncertainty presented with mean R^2^ values for each sex, with SD and range [27]. In this case, the mean (SD) R^2^ values were 0.89 (0.06), 0.90 (0.07), and 0.90 (0.07) for women, men, and all together, respectively. Uncertainty explained in this way is different than in ${\dot{V}}_{E}^{1}$ and cannot be compared. To obtain a better comparison of the ${\dot{V}}_{E}^{1}$ and ${\dot{V}}_{E}^{2}$ models, the supplementary data from Greenwald et al. [28] were used to calculate minute ventilation with the ${\dot{V}}_{E}^{2}$ model Equation (3) and compare it with the measured minute ventilation values in the Supplementary Data. The same statistical measures were calculated for ${\dot{V}}_{E}^{2},\;$as they were for ${\dot{V}}_{E}^{1}$. The SAT uncertainty was also incorporated in the overall uncertainty of the model.

${\dot{V}}_{E}^{3}$ uses data from tables provided by the EPA for certain age groups, which includes mean minute ventilation values and some specific percentiles, such as the 5th and 95th percentile. The difference between these two values provides a 90% confidence interval for the values used in this model. Table 1 shows the percentiles of minute ventilation for P1 and P2 participants involved in this research. The difference between the mean and the percentile is slightly larger at the 95th percentile than at the 5th percentile, with the average value being 34% for the 95th and 30% for the 5th percentile. The overall uncertainty estimate was determined as the mean of all the differences with SD.

Although ${\dot{V}}_{E}^{4}$ uses a similar approach as ${\dot{V}}_{E}^{3}$, the uncertainty is different as the “share of the day” values also have 5th and 95th percentile values and are not definitive, as in ${\dot{V}}_{E}^{3}$. As shown in Table 2, the differences between the 5th and 95th percentiles and the mean vary quite substantially between lower and higher intensity activities, with an average of 12% (14% for P2) in the “sedentary and passive” category, and 121% (132% for P2) in the “high intensity” category. Each uncertainty interval was weighted by the percentage of the day it represents, in contrast to the percentage it “should” represent, which in this case is ¼. To assess the final uncertainty for ${\dot{V}}_{E}^{4}$, the uncertainty from minute ventilation, as shown in Table 2, must be added by the method used in Equation (5).

As calculating the intake dose is a product of PM concentration values and calculated minute ventilation values, the uncertainty propagation is calculated by the method described in Equation (5).

All the calculations and visualizations were made in R v3.61 [41].

## 3. Results

### 3.1. Results of the Collocation

**Figure 1:**
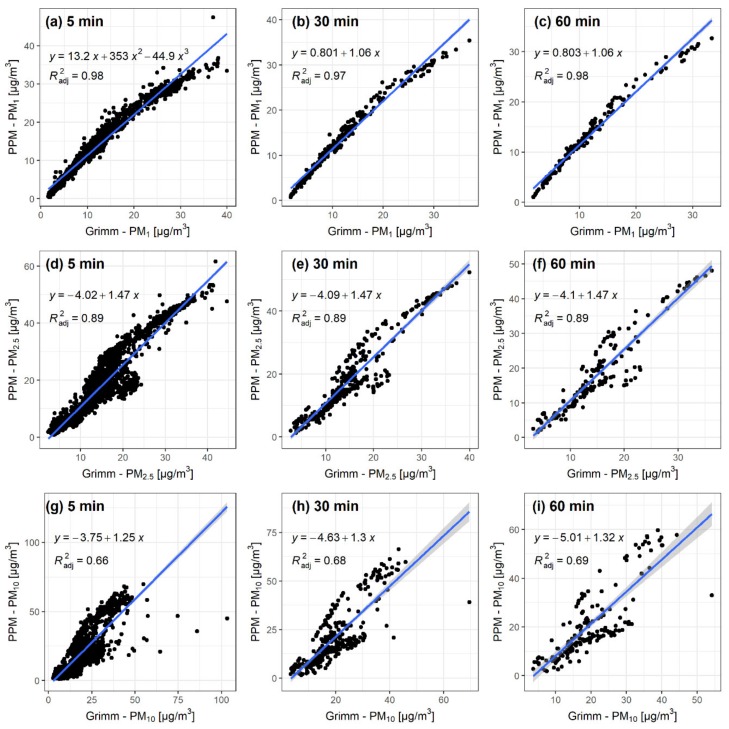
Correlation plots from collocating the portable Arduino based low-cost particulate matter (PM) measuring (PPM) unit with GRIMM. Rows present different sizes of particulate matter (PM_1_ (**a**–**c**), PM_2.5_ (**d**–**f**), PM_10_ (**g**–**i**)) and columns different time intervals (5 min (**a**,**d**,**g**), 30 min (**b**,**e**,**h**), 60 min (**c**,**f**,**i**)).

Figure 1 shows the correlation plots between GRIMM and PPM for the collocation with three different time intervals (5, 30, and 60 min) and all three particle size classes (1, 2.5, and 10 µm). Increasing the particle size reduces the linearity of the data points along the linear model regression line, which is also apparent in the R^2^ values that start at ~0.97 for PM_1_, drop to ~0.89 for PM_2.5_, and further drop down to ~0.68 for PM_10_ particles. As presented in Table 3, R^2^ values slightly increase with larger time intervals, as evident with PM_1_ particles (from 0.97 to 0.98) and with PM_10_ particles (from 0.66 to 0.69). These increases are relatively minimal and counteract the increase in the confidence interval with larger time intervals. Similarly, as in the case of R^2^, RMSE values increase as the size of the particles increases.

Mean (min–max) concentrations recorded for the PPM were 13.2 (0.4–47.4) µg/m^3^, 18.4 (0.8–61.6) µg/m^3^, and 20.6 (1.0–69.8) µg/m^3^ for PM_1_, PM_2.5_, and PM_10_, respectively, while the mean (min–max) GRIMM values were 11.7 (1.6–40) µg/m^3^, 15.2 (2.4–44.6) µg/m^3^, and 19.4 (2.7–103.4) µg/m^3^ for PM_1_, PM_2.5_, and PM_10_, respectively. These numbers generally coincide with measurements from the government-run AQ station in Ljubljana, which showed an average concentration of 11.4 µg/m^3^ for PM_2.5_ and 18.9 µg/m^3^ for PM_10_ [42,43] for the same time period. For PM_2.5_, the PPM device measured 7.0 µg/m^3^ higher average values than the AQ station, and the GRIMM device 3.8 µg/m^3^ higher values. For PM_10_, these values were 1.7 µg/m^3^ higher for the PPM, and 0.5 µg/m^3^ higher for GRIMM. As the distance to the AQ station was 2 km, some deviation is expected and these numbers fall in this range of expectations.

Although the PPM unit was validated for 5-, 30-, and 60-min intervals, intraclass differences of R^2^ and RMSE values varied less than interclass differences. The uncertainty associated with 1-min values for each size class should, therefore, be similar to the values calculated for 5-min averages. These results show that the PM_1_ values with the highest possible temporal resolution (1-min) have the least uncertainty, and are used to calculate intake doses.

### 3.2. Intake Dose Results

**Figure 2:**
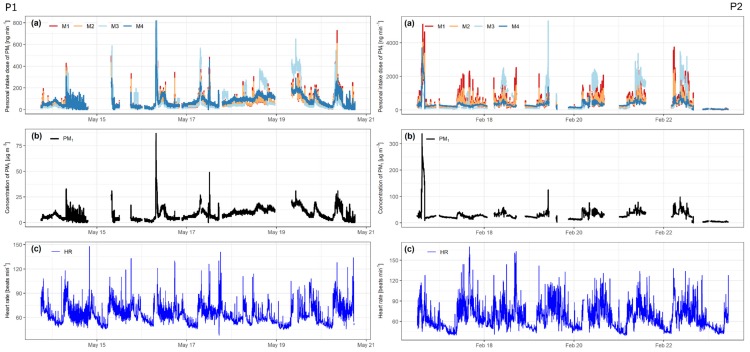
(**a**) Calculated intake dose of PM_1_ for all four models, (**b**) measured concentrations of PM_1_, and (**c**) heart rate in beats per minute. Left side for participant 1 (P1) and right side for participant 2 (P2).

The results of the calculations, based on all four intake dose models, are shown in Figure 2, plot (a), accompanied by plotted PM_1_ values for each participant in plot (b) and HR values in plot (c).

As evident in plot (b), the PM_1_ concentrations were higher for P2 than for P1. This is also evident in Table 4, where the summary statistics for PM_1_ concentrations show higher numbers for P2. The mean PM_1_ values for P1 and P2 were 8.1 µg/m^3^ and 28.6 µg/m^3^, respectively, which is more than a three-fold difference, and the maximum PM_1_ values were 87.0 µg/m^3^ and 338.0 µg/m^3^ for P1 and P2, respectively. The measured PM_2.5_ values were in the same range as values reported by the government-run AQ station in Ljubljana. Summary statistics for HR show that the maximum and standard deviation (SD) values were higher for P2, but the differences are not as pronounced as with PM concentrations.

**M1** and **M2** intake dose assessments show a strong relationship. As evident in Table 4, both have similar descriptive statistics, except the maximum intake dose value, which is noticeably higher for M1 with both P1 and P2.

**M3** follows a somewhat similar pattern as M1 and M2, although some deviations are evident. For P1, M3 mean value is ~15% higher than that of M1 and M2; the SD is almost double; and the maximum value is 11% and 26% higher than M1 and M2, respectively. The median and the Q1 and Q3 values are 10–30% lower. Similar ratios are found for P2, except the median value is almost half that of M1 and M2.

**M4** shows noticeably different results for P1 and P2. As evident from plot (a) in Figure 2, the results of M4 for P1 mostly follow the same trend as the other models. The summary statistics for M4, shown in Table 4 (for P1), show somewhat higher values than those of M1 and M2. For P2, as shown in Figure 2, the M4 results do not follow the trend of the doses based on other models as well as for P1. Although the mean, SD, and Q3 values are lower than in the other three, the median is almost the same (262.6 ng/min for M1 and M2, and 263.3 ng/min for M4).

### 3.3. Results of Quantifying Uncertainty

All the sensors used in this research have some uncertainty in their measurements, which is carried on to minute ventilation and intake dose calculations.

#### 3.3.1. Uncertainty in PM and Heart Rate Sensors

The results for the statistical measures of agreement with the reference data calculated for the PPM were as follows: R^2^ = 0.97, MAPE = 15.62%, MAE = 1.66, RMSE = 2.15, and MAE% = 14.17%. After including the uncertainty (listed as reproducibility) of the GRIMM, the overall uncertainty of the **PPM** is ~16%, which is estimated with the uncertainty propagation Equation (5).

The results from Oniani et al. [37] show that there is some disagreement between the **SAT** devices and the ECG. MAPE values ranged from 4.34% to 16.00% (mean: 9.82%, median: 8.85%, SD: 3.56%), and ICC from 0.91 to 0.02 (mean: 0.67, median: 0.71, SD: 0.25).

#### 3.3.2. Uncertainties for Minute Ventilation Models

${\dot{V}}_{E}^{1}$ statistical measures of agreement with the reference data were as follows: R^2^ = 0.82, MAPE = 28%, MAE = 6.47, and RMSE = 9.41. Combining the mean value of the SAT uncertainty (9.82% ± 3.56%; weighted: 9.82% ± 3.56% ∗2.39=24% ± 9%), the mean value of the FVC 90% confidence interval (~22% ± 2%; weighted: 22% ± 2% ∗0.52=11% ± 1%) and the MAPE of the model, using the approach described in Equation (5), yields an estimate of overall uncertainty for ${\dot{V}}_{E}^{1}$ of ~38% ± 9%.

${\dot{V}}_{E}^{2}$ calculated statistical measures were as follows: R^2^ = 0.72, MAPE = 32%, MAE = 7.68, and RMSE = 11.68. Adding the uncertainty in the SAT (9.82% ± 3.56%, adjusted to 9.82% ± 3.56%∗e=27% ± 10%) to the 32% uncertainty in the model gives an estimate of the uncertainty of ~42% ± 10% for ${\dot{V}}_{E}^{2}$.

${\dot{V}}_{E}^{3}$ uncertainty estimate was determined to be ~35% ± 7%, which can be considered an overall uncertainty value, in this case, for a 90% confidence interval.

${\dot{V}}_{E}^{4}$ uncertainty for the “share of day” variable is ~30% ± 16% for a 90% confidence interval. The final average value with SD for overall uncertainty estimate, with minute ventilation uncertainty included, is calculated to ~46% ± 17% for a 90% confidence interval.

#### 3.3.3. Propagation of Uncertainty

By adding the uncertainty from the PPM device, the final uncertainties for the intake dose assessment are 41% ± 9% and 45% ± 10%, for intake dose assessment models **M1** and **M2**, respectively, and 38% ± 7% and 49% ± 17% for **M3** and **M4**, respectively.

## 4. Discussion

Collocating the PPM with the GRIMM showed that the low-cost sensor provides valid data. These results showed that the sensor is fit for purpose, especially if the results of the smallest particles measured (PM1) are considered.

Two participants were chosen to avoid skewed results. Most of their characteristics (age, height, sex, gender, nature of their work, sampling season) were different enough to enable an indication of the model’s response to the variation of respective input variables.

M1 and M2 intake dose assessment models show a strong relationship (Figure 2), deviating mostly in peak concentrations, where M1 predicts a higher intake dose than M2. This is more evident with P2, where the calculated intake dose is higher and the peaks are more pronounced. Interestingly, although M1 uses more variables and was determined based on a larger number of participants in multiple research than M2, they show similar results. M1 shows a greater response to higher concentrations of PM than M2. M1 showed the highest intake dose to be 17% and 21% higher with P1 and P2, respectively, than M2. P2 was exposed to PM_1_ concentrations more than three-times higher than P1, and the difference in M1 and M2 rose. This indicates that M1 is more sensitive to elevated concentrations, which could be a crucial aspect when determining the acute intake dose.

There are more peaks, which are more pronounced in the M3 intake dose assessment model, but the median values indicate that most of the calculated values are lower than in M1 and M2. Although, the weekly intake doses do not differentiate much between the first three dose assessments and are all around 0.59 mg for P1 and between 2.39 mg and 2.76 mg for P2. Calculating the intake dose on a larger time interval would be as good with M3 as with M1 or M2. The issue, in this case, would be the exaggerated response of the model to elevated concentrations. A better realignment of the calculated values in M3 to M1 and M2 could be possible with a different interpretation of the TAD and categorizing each activity more in line with the HR associated with it.

M4 intake dose assessment model results show that M1 and M2 are influenced by the HR variable and do correspond to changes, most notably in time intervals with elevated HR and concentrations of PM, while M4 does not. As it is only an “adjusted” PM value, meaning that all the PM measurements are multiplied with the same value, it is not influenced by the higher HR differences, present with the results from P2.

Uncertainty is associated with all stages of calculating intake dose of PM. There is inherent uncertainty in the GRIMM, PPM, and Garmin devices, and uncertainty that comes from calculating minute ventilation from HR, and all the generalizations associated with it. Using different models, published in individual papers, shows that presenting uncertainty is not uniform in this field. Papers describe the relationship between modelled and measured data with different statistical measures, which makes assessing uncertainty difficult and sometimes incomparable. Although uncertainties calculated for each minute ventilation model are not entirely comparable, the uncertainties for ${\dot{V}}_{E}^{1}$ and ${\dot{V}}_{E}^{2}$ can be compared separately, as can those for ${\dot{V}}_{E}^{3}$ and ${\dot{V}}_{E}^{4}$.

Calculated statistical measures for ${\dot{V}}_{E}^{1}$ and ${\dot{V}}_{E}^{2}$ show that ${\dot{V}}_{E}^{2}$ has poorer agreement with the reference data (lower R^2^ value) and higher errors (higher MAPE, MAE, and RMSE). The model with more variables (${\dot{V}}_{E}^{1}$) can calculate data that are closer to the measured data for minute ventilation. Both models have their uses, and although ${\dot{V}}_{E}^{2}$ has poorer results of calculated statistical measures than ${\dot{V}}_{E}^{1}$, it requires less information about the participant. Relatively high standard deviations in the uncertainty (~¼ of the value) show that the real uncertainty of the models is even closer than the uncertainty values themselves would suggest.

Because the uncertainties for ${\dot{V}}_{E}^{3}$ and ${\dot{V}}_{E}^{4}$ were calculated in the same manner, they can be compared. The results are as expected, where ${\dot{V}}_{E}^{4}$ has a higher uncertainty than ${\dot{V}}_{E}^{3}$ because the share of daily activity has a certain level of uncertainty, which is propagated to the minute ventilation estimate, which is also presented with an uncertainty interval.

The uncertainties calculated through a series of steps do provide some measure of the validity of each minute ventilation model. Each of the models was provided with a specific measure of agreement between the modelled and measured data, but as these measures were different, they are not entirely comparable. This was somewhat compensated with further calculations for ${\dot{V}}_{E}^{1}$ and ${\dot{V}}_{E}^{2}$. Further research is needed to validate each model directly with reference data.

## 5. Limitations

Collocating the PPM device took place in a room with open windows, so the environment was a mixture of outdoor and indoor, which is not the case during the deployment phase. Moreover, only one PPM device was evaluated during colocation with the reference instrument. To truly determine the validity of the sensor, more devices should be subjected to collocation for longer periods and in different seasons and conditions. The device was stationary for the entire period, which is not representative as the device is designed to be mobile. Indeed, most of the time, the device is stationary in real-life circumstances, for example, in the office, at home, in the bedroom, or in a car. The collocation of the device would be needed while it is mobile. Using the GRIMM device for the collocation was the only available option at the time, but in further validation of the PPM device, it should be compared to a certified government AQ station or similar.

Data from two participants were used for the models. There were certain differences between the participants, but they were also both Caucasian and had a similar height and weight. The latter is also discussed in the Supplementary Materials. For both participants, there were some data missing, and the TADs were somewhat incomplete or inconsistent. All of the models were also validated indirectly by validating the minute ventilation and particulate matter concentrations separately. Future research should develop methods for direct validation of the models, using real-time data with a high temporal resolution for each observed variable, with research-grade instruments.

Measures of uncertainty were provided for all minute ventilation models, but were inconsistent and not entirely comparable.

## 6. Conclusions

A comparison of the four different approaches to assess intake dose, using data from low-cost sensors, was presented. Collocating the PPM device with a more expensive, research-grade instrument showed that the sensor provides good data, and was reliable enough to use it to determine intake dose of PM. Agreement with the reference instrument was better with smaller-sized particles, but the differences for different time averaging intervals were only marginal (ΔR^2^ = 0.01–0.03). Considering these results, PM_1_ concentrations were used for modelling, with the highest temporal resolution possible (1 min).

Four different minute ventilation models with increasing levels of complexity were used to determine minute ventilation, which was then used to calculate the intake dose of PM. Intake dose assessment models M1 and M2, which used HR as a variable, showed good agreement with each other, although M1, which was more complex and used sex, age, height, weight, and FVC as variables, showed more pronounced peaks and a stronger response to elevated HR and PM concentrations than M2, which only used sex as a variable (apart from HR). Intake dose assessment M3 and M4 did not use HR as a variable, but relied on generalized population data for specific activities, differentiated by sex and age. M4 showed better agreement with M1 and M2 than M3, but this could be the result of inaccurate activity classification. With further optimization, M3 could be improved and better realigned with other models.

Comparing the uncertainties between all the minute ventilation models was not possible, owing to different measures of uncertainty being reported for each model. After some additional calculations, a direct comparison of ${\dot{V}}_{E}^{1}$ and ${\dot{V}}_{E}^{2}$ was possible and between ${\dot{V}}_{E}^{3}$ and ${\dot{V}}_{E}^{4}$. ${\dot{V}}_{E}^{1}$ had lower uncertainty than ${\dot{V}}_{E}^{2}$, which is mostly associated with the model itself and less with the SAT and other variables. The comparison of ${\dot{V}}_{E}^{3}$ and ${\dot{V}}_{E}^{4}$ showed that ${\dot{V}}_{E}^{3}$ had less uncertainty associated with it than ${\dot{V}}_{E}^{4}$, which was a direct consequence of ${\dot{V}}_{E}^{4}$ using another set of generalized population data to determine “share of the day” for each specific activity, for which ${\dot{V}}_{E}^{3}$ had data from TADs. The minute ventilation models contributed the largest share to the overall uncertainty of the intake dose assessment models, followed by the SAT and finally the PPM.

As evident in this work, there are several different approaches for calculating the intake dose of pollutants. This stems also from different goals that the developers of these models set out in their respective studies. While some validate existing models, others try to evaluate the models predicting ability. Future research can use these results to determine which model best suits their needs and resources. While more complex models provide dose calculations on a minute-by-minute basis and have less uncertainty, they also require more resources in terms of sensors used and invested time by the researchers and the participants. This paper can also provide several options for future research in PM intake dose assessment, from developing models with less uncertainty, using location data and different sensors, and using the described models on larger groups.

As low-cost sensor technology is rapidly developing, there is an ever-expanding field of possibilities of how to implement such technologies for sensing and intake dose assessments. To allow comparability between the results of these measurements and calculations, a more homogeneous approach to presenting these finding and the uncertainties associated with them is needed. This work is a contribution towards this goal—by using appropriate terms and methods, this paper will contribute to further developing a unified methodological and terminological approach in this type of research. Modelling the intake dose of PM, by determining certain variables with low-cost sensors, was shown to be possible. Although there are many advantages, there is uncertainty that comes with this kind of sampling, and researchers need to account for this aspect in reporting their data. As this technology and these approaches become more widespread and distributed in the general public, users must be made aware that these data can come with wide margins of uncertainty and should only be used as a general guideline and not a scientific fact.

## Figures and Tables

**Table 1:** Mean, 5th, and 95th percentile minute ventilation values for P1 and P2 with calculated differences between the percentiles and the mean, provided in the Environmental Protection Agency (EPA) Exposure Handbook [39].

	Activity	Mean [L/min]	5th % [L/min]	95th % [L/min]	$\;\frac{\left|5^{th}-mean\right|}{mean}\;$	$\;\frac{\left|95^{th}-mean\right|}{mean}\;$
**P1**	**Sedentary and Passive**	4.1	2.9	5.6	0.30	0.36
	**Light intensity**	10	7.4	13	0.25	0.30
	**Moderate intensity**	21	14	30	0.31	0.43
	**High intensity**	39	24	58	0.38	0.46
**P2**	**Sedentary and Passive**	4.4	2.9	5.3	0.35	0.22
	**Light intensity**	11	11	13	0.05	0.22
	**Moderate intensity**	24	15	32	0.36	0.37
	**High intensity**	43	27	58	0.36	0.36

**Table 2:** Mean, 5th, and 95th percentile daily share of activity for P1 and P2, with calculated differences between the percentiles and the mean, provided in the EPA Exposure Handbook [39].

	Activity	Mean [hours]	5th % [hours]	95th % [hours]	$\;\frac{\left|5^{th}-mean\right|}{mean}\;$	$\;\frac{\left|95^{th}-mean\right|}{mean}\;$
**P1**	**Sedentary and Passive**	13	11	14	0.12	0.12
	**Light intensity**	6.5	4.1	9.4	0.37	0.45
	**Moderate intensity**	4.6	1.7	7.1	0.63	0.56
	**High intensity**	0.3	0.03	0.9	0.91	1.5
**P2**	**Sedentary and Passive**	12	11	14	0.13	0.14
	**Light intensity**	5.7	2.8	10	0.51	0.83
	**Moderate intensity**	5.7	1.3	8.9	0.78	0.56
	**High intensity**	0.4	0.03	1.0	0.92	1.71

**Table 3:** Relationship between portable Arduino based low-cost particulate matter (PM) measuring (PPM) unit and GRIMM. RMSE, root mean square error.

PM class	Time	R^2^	RMSE	Intercept	Slope
PM1	5 min	0.97	2.15	0.83	1.06
	30 min	0.97	2.01	0.80	1.06
	60 min	0.98	1.96	0.80	1.06
PM2.5	5 min	0.89	6.30	−4.02	1.47
	30 min	0.89	6.17	−4.09	1.47
	60 min	0.89	6.11	−4.10	1.47
PM10	5 min	0.66	9.07	−3.75	1.25
	30 min	0.68	8.76	−4.63	1.30
	60 min	0.69	8.58	−5.01	1.32

**Table 4:** Descriptive statistics for intake dose assessments based on all four models, PM_1_ concentrations, and heart rate (HR) values for both participants. Recovery represents the percent of data recovered, where 100% is the entire period of observation. Sum represents the accumulated dose for the entire week of observation.

	Participant 1 (P1)					
	**M1**	**M2**	**M3**	**M4**	**PM1 [µg/m^3^]**	**HR [bpm]**
Mean	60.2	58.8	69.6	75.9	8.1	63.8
SD	58.9	53.1	99.6	55.3	5.9	11.9
Median	43.3	44.4	29.1	65.8	7.0	62.0
Q1–Q3	25.0–73.7	26.2–74.3	16.6–63.1	37.6–104	4.0–11.0	55.0–70.0
Min–Max	0.0–729	0.0–609	0.0–820	0.0–818	0.0–87.0	38.0–148
Recovery [%]	78.2	78.2	80.7	80.7	80.7	97.2
Sum	599,520	580,128	589,983	617,486	67,352	/
	**Participant 2 (P2)**					
	**M1**	**M2**	**M3**	**M4**	**PM1 [µg/m^3^]**	**HR [bpm]**
Mean	415	359	493	314	28.6	66.3
SD	476	359	698	280	25.5	18.2
Median	263	262	109	263	24.0	63.0
Q1–Q3	108–542	140–465	91.5–595	219–373	20.0–34.0	52.8–76.0
Min–Max	0.0–5110	0.0–4033	0.0–5313	0.0–3708	0.0–338	39.0–170
Recovery [%]	66.7	66.7	51.2	68.1	68.1	98.6
Sum	2,764,423	2,391,141	2,522,915	2,136,003	194,702	/

## Supplementary Materials

The following are available online at https://www.mdpi.com/1424-8220/20/5/1406/s1, Figure S1: Boxplots of intake dose calculations with different height and weight variations as 67 referenced in Table S3, Table S1 Basic specifications for GRIMM Model 11-A, Table S2: Excerpt from Plantower pms5003 datasheet with some relevant figures about the functionality of the sensor, Table S3: Matrix of all variations for four different weights and heights. The Supplementary Materials (SI) contain extended descriptions of the sensors used in this research, a more detailed overview of the data collection process, and a brief investigation of the influence of weight and height of the participant on the final results.
