# Supplementary material for: Comparing Airborne Particulate Matter Intake Dose Assessment Models Using Low-Cost Portable Sensor Data
Source: Sensors (Basel). 2020 Mar 4;20(5):1406. doi: 10.3390/s20051406 (PMC7085603; doi:10.3390/s20051406)
Supplement: Supplementary file 1 [file sensors-20-01406-s001.pdf]

1. Supplementary Information

Information About the GRIMM Device

The PPM unit was collocated with a GRIMM (Durag Group, Hamburg, Germany) Model 11-A (1.109) Aerosol Spectrometer (GRIMM), that was used as a research-grade reference instrument for PM measurements. Measurements, obtained with 90° light scattering and filter-sampling, of particle concentrations in 31 different channels were collected and summed to form the same three size channels as the data provided by the PPM unit. Some further specifications of the GRIMM are listed in **Table 1**.

Table 1. Basic specifications for GRIMM Model 11-A [44]

Parameter	Value
Range of measurement	0.25 µm–32 µm; 31 size channels
Particle concentration	1 to 3,000,000 particles/litre
Dust mass	0.1 to 100,000 $\mu g/m^3$
Volume flow	1.2 litre/minute
Reproducibility	5 % for the whole range
Light source	Diode-laser (λ = 683 nm, P_{max} = 40 mW)
Storage interval	1 min to 1h selectable
Sampling time	6 sec (normal), 1, 2, 3 sec (fast mode)

As referenced in the GRIMM manual, the reproducibility value is achieved by comparing the relative errors between the GRIMM device and a certified reference instrument, and calibrating it, until the readings of the comparison fall within ±2 % accuracy, which is set to ±5 % for the certified accuracy, due to the fact that particle diameter affects particle mass by the third power.

Office space at Jožef Stefan Institute, Ljubljana, Slovenia (LAT: 14.487854, LON: 46.042365) with open windows was used for the collocation to simulate outdoor conditions. The window was facing northwest by north and had a distance of around 65m from the road, with moderate to low traffic volume.

Information About the Ppm Device and Participant Instructions

. In addition to PM, ancillary parameters measured by the PM unit include temperature, relative humidity, the coordinates for the location of the device, the speed, altitude and a timestamp. The device is charged through a standard micro USB port and has a battery life of 6 to 7 hours. Data retrieval is possible in two ways:

- through a wireless mobile network on a dedicated IoTech server, or
- manually downloading the data, stored on an SD card.

Selected characteristics, provided by the manufacturer of Plantower pms5003 sensors, are presented in **Table 2**.

Table 2. Excerpt from Plantower pms5003 datasheet with some relevant figures about the functionality of the sensor [35]

Parameter	Value
Range of measurement	0.3 µm–1.0 µm; 1.0 µm–2.5 µm; 2.5 µm–10 µm
Counting efficiency	50% @ 0.3µm; 98% @ >=0.5µm
Range	0~500 $\mu g/m^3$
Resolution	1 $\mu g/m^3$
Max consistency error ($PM_{2.5}$)	±10% @ 100~500$\mu g/m^3$<br>±10$\mu g/m^3$ @ 0~100$\mu g/m^3$

The participants carried the PPM device with them the entire week. Not necessarily on them, but at least nearby. The device has a clip affixed on the side, so the person could choose to wear it on their belt, handbag or backpack. A USB charger was provided with instructions to charge the device whenever they can, not to wait for the battery to be depleted. After the end of the sampling week, the top side of the housing was removed and the data was manually copied from the SD card as a safety measure.

Measuring Heart Rate and Physical Activity

Continuous HR measurements were made by using a commercial Smart Activity Tracker (SAT), a Vívosmart 3 from Garmin International [36]. The device is a digital smartwatch and is waterproof and resistant to dust. It has a battery life of around 4 to 5 days and can store 14 days of data. To export the data the device must be connected by cable to a laptop or a smartphone via a Bluetooth connection.

Research participants were instructed to keep the device on their wrists the entire week, 24 hours a day, except for about two hours when the device needed to be charged.

Data storage took place on a server run by Garmin, but the data was not available in raw format to be used in the analysis. An ICARUS specific data portal was established for the project where the SAT data was transferred and later downloaded in raw form (.csv files). The data was in minute temporal resolution and provided values for heart rate, specific physical activity, steps taken, calories "burned", distance walked and stress.

Iterations of M1 with Modified Variables

To investigate the influence of weight and height on M1, and its comparison to M2, some iterations were made with modified weight and height variables for P1. All possible variations of four different weights and heights were explored, as seen in the matrix, presented in **Table 3**, with intake dose results being abbreviated with "pEx". Descriptive statistics of the results of these calculations are shown in **Figure 1**, with the highest median value (Q1, Q3) being 49.97 (28.82, 85.02) ng/min in pEx8 and the lowest 39.63 (22.86, 67.43) ng/min in pEx13, compared to the original calculation pEx0 43.29 (24.96, 73.65) ng/min, and the calculation made with M2, pEx_M2 44.39 (26.18, 74.27) ng/min.

These results show that the weight and height factors do influence intake dose calculations, relative to M2, where sex is the only factor used, apart from heart rate, which remained the same in all calculations. Although, the average median of all the different calculations is 44.65 (25.75, 75.97) ng/min, which is close to the pEx_M2 median of 44.39 (26.18, 74.27) ng/min.

Table 3. Matrix of all variations for four different weights and heights

	**Weight [kg]**			
**Height [cm]**	**45**	**55**	**65**	**75**
**155**	pEx1	pEx5	pEx9	pEx13
**165**	pEx2	pEx6	pEx10	pEx14
**175**	pEx3	pEx7	pEx11	pEx15
**185**	pEx4	pEx8	pEx12	pEx16

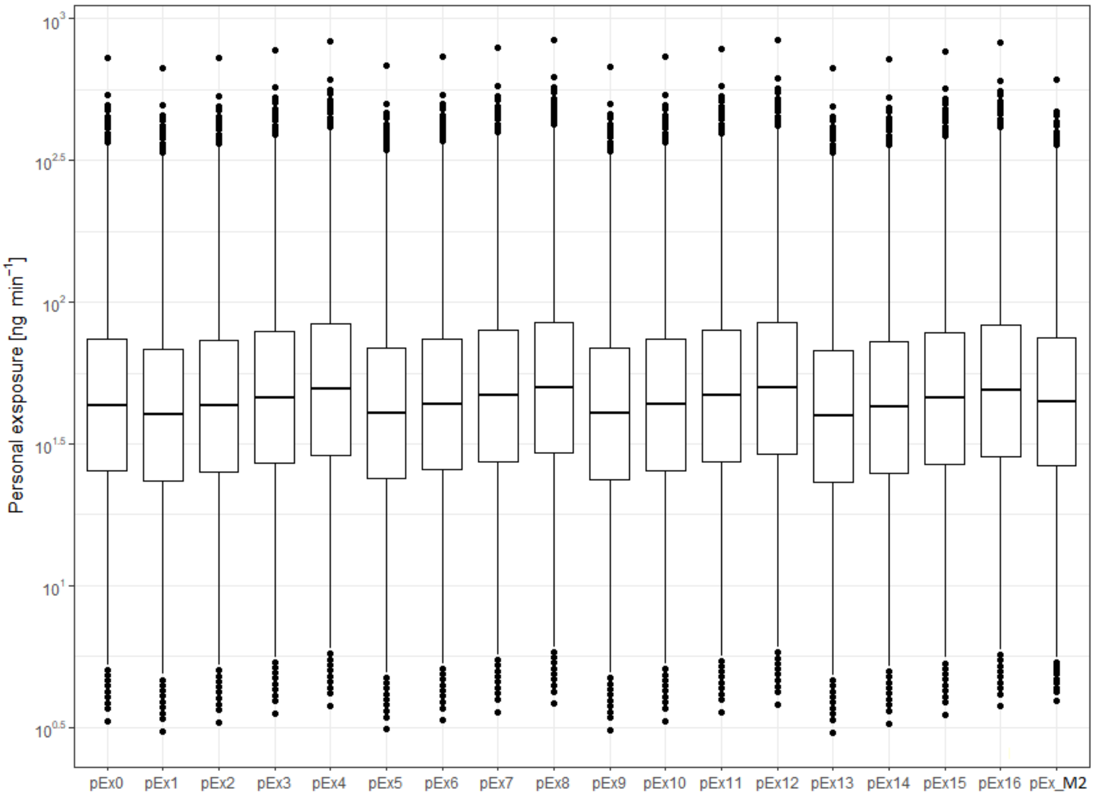

Figure 1: Boxplots of intake dose calculations with different height and weight variations as referenced in **Table 3**, pEx0 being the intake dose results of the participant in this research. The values have been logarithmically transformed.
